# Concomitant Small Cell Neuroendocrine Carcinoma of Gallbladder and Breast Cancer

**DOI:** 10.1155/2014/945921

**Published:** 2014-09-25

**Authors:** Paolo Aiello, Francesco Aragona, Valentina Territo, Anna Maria Caruso, Rosalia Patti, Salvatore Buscemi, Gaetano Di Vita

**Affiliations:** ^1^Dipartimento di Discipline Chirurgiche, Oncologiche e Stomatologiche, Unità di Chirurgia Generale, Università degli Studi di Palermo, Via Liborio Giuffrè No. 5, 90127 Palermo, Italy; ^2^Dipartimento di Patologia Umana, Facoltà di Medicina e Chirurgia, Policlinico Universitario, 90127 Palermo, Italy

## Abstract

The neuroendocrine carcinoma is defined as a high-grade malignant neuroendocrine neoplasm arising from enterochromaffin cells, usually disposed in the mucosa of gastric and respiratory tracts. The localization in the gallbladder is rare. Knowledge of these gallbladder tumors is limited and based on isolated case reports. We describe a case of an incidental finding of small cell neuroendocrine carcinoma of the gallbladder, observed after cholecystectomy for cholelithiasis, in a 55-year-old female, who already underwent quadrantectomy and sentinel lymph-node biopsy for breast cancer. The patient underwent radiotherapy for breast cancer and six cycles of chemotherapy with cisplatin and etoposide. Eighteen months after surgery, the patient was free from disease. Small cell neuroendocrine carcinoma of the gallbladder has poor prognosis. Because of the rarity of the reported cases, specific prognostic factors have not been identified. The coexistence of small cell neuroendocrine carcinoma of the gallbladder with another malignancy has been reported only once. The contemporary presence of the two neoplasms could reflect that bioactive agents secreted by carcinoid can promote phenotypic changes in susceptible cells and induce neoplastic transformation.

## 1. Introduction

Neuroendocrine tumors (NETs) are rare neoplasms, arising from enterochromaffin cells, usually disposed in the mucosa of gastric and respiratory tracts. Neuroendocrine cells have some biochemical and morphological characteristics: these cells contain dense core secretory granules and lack axons and synapses and are able to produce neurotransmitters, neuromodulators, or neuropeptide hormones. Incidence of NETs is 5.25 cases per 100,000 person-years in the United States [1]. NETs most frequently arise in the gastrointestinal tract and in the respiratory tract secondarily but can occur in any location. According to the data collected by the Surveillance, Epidemiology, and End Results (SEER) program of National Cancer Institute of the USA in the period of 1973–2005 using SEER 17 Registry, of 16005 of NETs of the entire gastrointestinal system only in 229 cases (1.4%) was the origin of the gallbladder [2].

The NETs comprise a heterogeneous group of neoplasms that vary from low-grade malignancy tumors to tumors with high malignancy. A recent classification published in 2010 by the World Health Organization divides NETs into three categories [3]: neuroendocrine tumor (NET) which includes classic carcinoid tumor, neuroendocrine carcinoma (NEC), and mixed adenoneuroendocrine carcinoma (MANEC). A NEC is defined as a high-grade malignant neuroendocrine neoplasm composed of either small or intermediate to large cells with marked nuclear atypia and a high proliferation fraction.

NETs have a good prognosis, while NEC and MANEC have a poor prognosis. Incidence of various types of NETs in the entire gastrointestinal system is different; however, the ratio of tumor with good prognosis to tumor with severe prognosis in the gallbladder is very low in comparison to other gastrointestinal locations [2]. Because of rarity of NEC of gallbladder, knowledge of these tumors is limited and based on isolated case reports or very small series.

We describe a case of incidental small cell NEC of the gallbladder (NEC-SC-GB) concomitant with moderately differentiated invasive ductal carcinoma of the breast. To the best of our knowledge, this is the first case that describes this association.

## 2. Case Presentation

A 55-year-old female, with symptoms related to cholelithiasis, was admitted to our hospital. She reported a 20-day history of intermittent right upper quadrant pain radiating to the back, associated with nausea and bloating sensation. Her family history included father with lung adenocarcinoma. Her personal history included right ovary salpingectomy for endometriosis cyst, viscerolysis for bowel obstruction caused by adhesions, and repair of a median abdominal incisional hernia using polypropylene mesh. A quadrantectomy and sentinel lymph-node biopsy for moderately differentiated invasive ductal carcinoma of the breast G2pT1cN0, with estrogens receptors of 90%, progesterone receptors of 85%, and HER2/neu = 1+ and Ki-67 < 14%, was performed one month before. At the time of admission, she had normal vital parameters and laboratory investigations. At the physical examination, tenderness in epigastric region and in the right upper quadrant was revealed with no evidence of jaundice. The patient showed an abdominal ultrasound and contrast-enhance abdominal computed tomography: lumen of the gallbladder was occupied by numerous stones with a nonthickened wall, no evidence of biliary dilatation was noted, and there was no ascites. The chest X-ray revealed no unusual findings. After a laparoscopic access, we carried out, for the presence of numerous adhesions, an open cholecystectomy. There was no evident locoregional lymphadenopathy.

**Figure 1:**
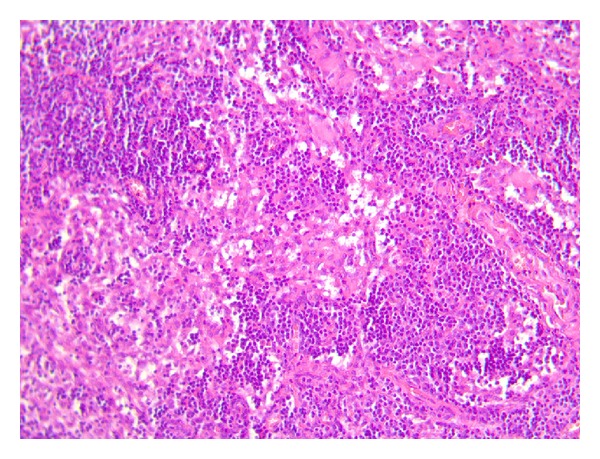
Focal metastatic colonization of the pericystic lymph node.

**Figure 2:**
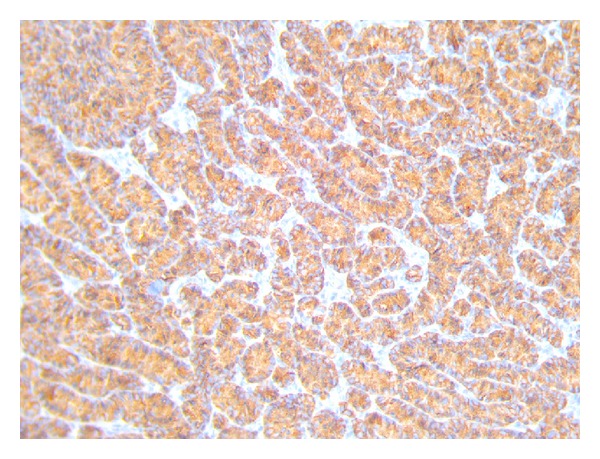
Positivity of the tumor to the immunohistochemical staining for chromogranin.

**Figure 3:**
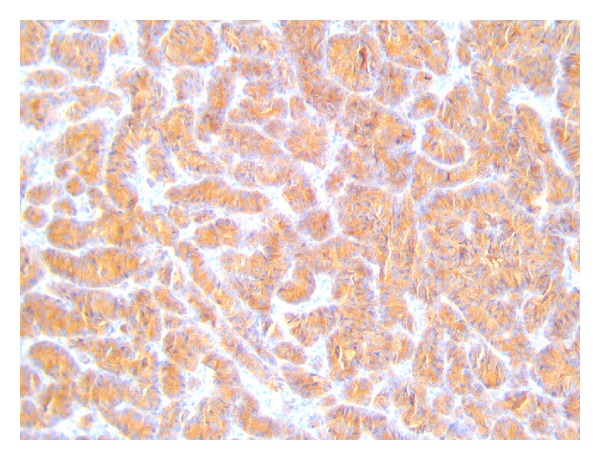
Positivity of the tumor to the immunohistochemical staining for synaptophysin.

The patient had an uneventful postoperative recovery and was discharged home after two days. On gross inspection, the gallbladder measured 10 cm in length, some stones were found in its lumen, and a polypoid, whitish lesion, in correspondence with the bottom, measuring 1,5 cm was found in its body. Final histologic examination revealed a neuroendocrine small cell carcinoma of the gallbladder. Tumour was seen infiltrating into the muscular layer but not through the serosa of the gallbladder. Resected margins were free from tumor. Two lymph nodes measuring about 4 mm were isolated from the surgical specimen: the analysis of one lymph node revealed focal metastatic deposits of neoplastic cells (Figure 1), whereas in the other one no deposits of tumor cells were found. Immunohistochemical studies revealed cells strongly positive for chromogranin (Figure 2) and synaptophysin (Figure 3) and negative for thyroid transcription factor-1.

Postoperative imaging studies did not show residual disease either locally or remotely. Patient was subjected to radiotherapy for breast cancer and six cycles of chemotherapy with cisplatin and etoposide were administered. Eighteen months after surgery, the patient was free from disease.

## 3. Discussion

NEC-SC-GB was described by Albores-Saavedra et al. in 1981 [4]. Subsequently, only a case report or small series of patients have been published [5]; however, a paucity of data on this tumor persists; Mahipal and Gupta [6] reported that 74 cases have been described until 2011.

Hystogenesis of NEC-SC-GB is unclear. Some authors [7] believe that it arises from rare neuroendocrine cells normally present in the body and fundus of the gallbladder. Other authors believe that it originates from foci of intestinal or gastric metaplasia [8], usually associated with long-standing chronic inflammation due to cholelithiasis [9]. The age at presentation has a range from 38 to 81 years with a higher incidence in the sixth and seventh decade of life; the female to male ratio is 2.2 [2].

Often stones are associated with the NEC-SC-GB, while rarely it presents as an isolated lesion wall [10]. Tumor size ranges from 0.4 cm to over 10 cm. The symptomatology has no pathognomonic characters but is similar to that of lithiasis in the early stage NEC-SC-GB and in the later stages is similar to that of adenocarcinoma.

The secretory activity characteristic of NETs has rarely been observed in NEC-SC-GB and in this case, the tumor was in an advanced stage. Cases with symptoms of ghrelin secretion [11] of insulin [12], of pancreatic polypeptide [13], of paraneoplastic sensory neuropathy [14], or of hyponatremia [15] have been described.

These tumors are usually diagnosed incidentally at the histological examination of gallbladder specimens after cholecystectomy, during autopsy, or during an ultrasound examination or computed tomography performed for other reasons [16]. In cases of multiple cholelithiases we should pay particular attention to imaging investigations because calculi can disguise cancer as we observed.

Coexistence of malignant tumors of different histologic types with carcinoids has been a source of significant debate, throughout the past several decades [17]. A synchronous or metachronous neoplasia with carcinoid develops in approximately 25% of cases [17, 18]. These lesions are located more frequently in the gastrointestinal tract. An explanation for the high frequency is not clear. It presumably reflects the fact that some of the bioactive agents secreted by these lesions are known mitogens for a variety of cell types. Therefore, it is probable that, over time, the prolonged action of such growth factors may promote phenotypic changes in susceptible cells and induce neoplastic transformation. To the best of our knowledge, only Petersen et al. [19] reported a case of a 73-year-old woman with NEC-SC-GB concomitant with an endometrioid adenocarcinoma of the ovary. Therefore, the case that we observed represents the second case of multiple tumors reported in the literature.

Simple cholecystectomy is probably an adequate therapy for early detected and preinvasive cancer, while, for advanced neoplasms, a more aggressive radical surgical treatment, including radical cholecystectomy and regional lymphadenectomy combined with a hepatic resection in order to obtain adequate free margins, is needed [9].

Role of radiotherapy and chemotherapy remains undefined because of the paucity of data of this rare disease. There is no standard indication for adjuvant therapy, so decisions on adjuvant therapy are based on clinical situations, such as dissemination state, or on characteristics of the patients such as old age, the presence of medical comorbidities, or patient's refusal.

Prognosis of this pathology is worse due to the advanced nature of the disease at diagnosis [6], tumor size being often greater than 4 cm, and also the majority of patients presenting with liver metastases or lymph nodes or with hepatic infiltration.

A clear assessment of prognosis of NEC-SC-GB is difficult due to limited knowledge, because cases described have a limited follow-up. Kim et al. [5] and Eltawil et al. [9] reported in a series of cases of NEC-SC-GB collected by SEER that the 5-year relative survival was 0%, despite the fact that at least two patients had tumor size <2 cm [9]. Mahipal and Gupta [6] in the review of literature report a median survival of 9 months with a range of 1–189 months. The few reported cases with tumors of limited size [10, 20] have a limited follow-up and were detected as incidental findings.

## 4. Conclusion

NEC-SC-GB is a rare malignancy with poor prognosis. Because of rarity of reported cases specific prognostic factors have not been identified, but high tumor size, depth of invasion, and metastases are probably associated with the prognosis. Our case has not progressed 18 months after surgery, suggesting that aggressive multimodal treatment may be helpful.

We emphasize the need for surgical treatment of any wall thickening or polypoid lesion of the gallbladder in elderly women, relying on the pathologist and immunohistochemistry analyses for the final diagnosis. However, presence of gallstones can mask the tumor. Finally, although it has been reported only two cases of multiple tumours, this possibility should not be overlooked.
